# Advancements in Hematologic Malignancy Detection: A Comprehensive Survey of Methodologies and Emerging Trends

**DOI:** 10.1155/tswj/1671766

**Published:** 2025-05-18

**Authors:** Rajashree Nambiar, Ranjith Bhat, Balachandra Achar H V

**Affiliations:** ^1^Department of Robotics and AI Engineering, NMAM Institute of Technology, NITTE (Deemed to be University), Nitte, India; ^2^Department of Electronics and Communication Engineering, Manipal Institute of Technology Bengaluru, Manipal Academy of Higher Education, Manipal, India

## Abstract

The investigation and diagnosis of hematologic malignancy using blood cell image analysis are major and emerging subjects that lie at the intersection of artificial intelligence and medical research. This survey systematically examines the state-of-the-art in blood cancer detection through image-based analysis, aimed at identifying the most effective computational strategies and highlighting emerging trends. This review focuses on three principal objectives, namely, to categorize and compare traditional machine learning (ML), deep learning (DL), and hybrid learning approaches; to evaluate performance metrics such as accuracy, precision, recall, and area under the ROC curve; and to identify methodological gaps and propose directions for future research. Methodologically, we organize the literature by categorizing the malignancy types—leukemia, lymphoma, and multiple myeloma—and particularizing the preprocessing steps, feature extraction techniques, network architectures, and ensemble strategies employed. For ML methods, we discuss classical classifiers including support vector machines and random forests; for DL, we analyze convolutional neural networks (e.g., AlexNet, VGG, and ResNet) and transformer-based models; and for hybrid systems, we examine combinations of CNNs with attention mechanisms or traditional classifiers. Our synthesis reveals that DL models consistently outperform ML baselines, achieving classification accuracies above 95% in benchmark datasets, with hybrid models pushing peak accuracy to 99.7%. However, challenges remain in data scarcity, class imbalance, and generalizability to clinical settings. We conclude by recommending the integration of multimodal data, semisupervised learning, and rigorous external validation to advance toward deployable diagnostic tools. This survey also provides a comprehensive roadmap for researchers and clinicians striving to harness AI for reliable hematologic cancer detection.

## 1. Introduction

Human blood plays a vital role in delivering oxygen throughout the body [1]. In the absence of a sufficient volume of blood, the body cannot obtain oxygen, which may even lead to death. There are four components in human blood [2], namely, red blood cells (RBCs), white blood cells (WBCs), platelets, and plasma, a transparent yellow liquid that includes hormones, electrolytes, minerals, proteins, and other chemicals necessary for the well-being of the human body. It accounts for over half of the blood volume.

More importantly, the bone marrow produces three distinct types of blood cells:

• Platelets, which aid in the clotting of blood and, as a result, prevent bleeding after a wound,
• RBCs or erythrocytes, which are widely distributed throughout the human body, and
• WBCs or leucocytes, which help the human body combat infections [3].

Leukocytes are further categorized into five subtypes based on morphology and function:

• Neutrophils: multilobed nuclei with granular cytoplasm—key to bacterial defence.
• Lymphocytes: round nuclei with scant cytoplasm—involved in adaptive immunity.
• Monocytes: large, kidney-shaped nuclei—precursors to macrophages.
• Eosinophils: bilobed nuclei with red-staining granules—involved in parasitic infections and allergic responses.
• Basophils: Granules obscuring the nucleus—play a role in hypersensitivity reactions.

**Figure 1:**
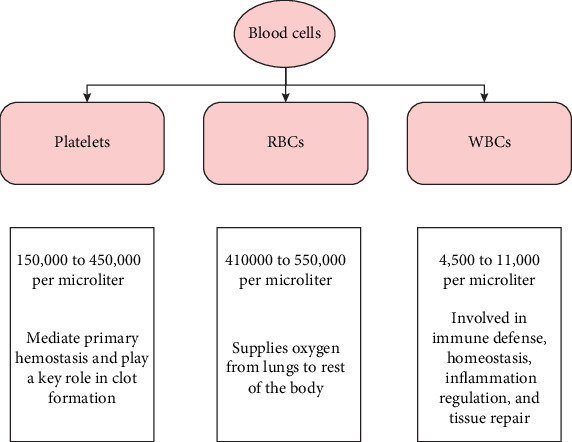
Classification and role of various blood cell types.

**Figure 2:**
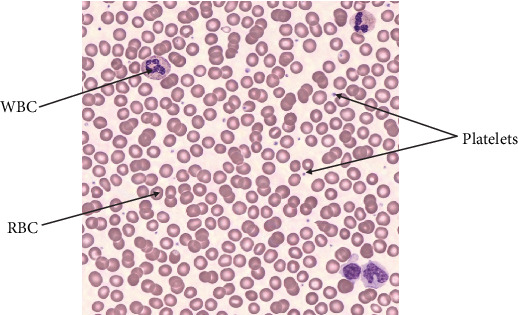
Normal blood sample [4].

Various blood cell types are displayed in Figure 1, and a normal blood sample is shown in Figure 2.

Blood cells are generated in the bone marrow and are consistently discharged into the bloodstream throughout the body. In hematologic malignancies, the normal hematopoietic process is significantly impaired due to the proliferation of abnormal blood cells. The unregulated multiplication of these cells considerably affects the development of functional blood components, but it is not the fundamental causal factor. The genesis of hematopoietic malignancies encompasses a complex interaction of genetic, epigenetic, and environmental variables that promote the emergence of these aberrant cells. In-depth examination of these fundamental factors is crucial for comprehending the origins of hematologic malignancies. Three of the most prevalent forms of blood cancer are leukemia and lymphoma. Blood cancer may arise due to genetic mutations within the DNA of hematopoietic stem cells, leading to dysregulated cellular proliferation and differentiation. It is because of these factors that the blood cells begin to behave irregularly. There is a strong correlation between these shifts and factors that are beyond our ability to detect the abnormalities. These are nongenetic anomalies that arise during an individual's lifetime and may affect future generations through methods including epigenetic alterations. Although these modifications do not entail changes in the DNA sequence, they can influence gene expression and may be inherited by progeny under specific circumstances. Over 40,000 people in the United Kingdom are diagnosed with blood cancer every year, and more than 250,000 people are actively coping with the conditions associated with blood cancer [5]. Worldwide, cancer is one of the leading killers within a wide range of age groups. Cancer is one of many diseases under investigation to gain control over illness. It is essential to recognize that a diverse range of tumors results from the unchecked proliferation of abnormal cells within the body. These cells have the potential to metastasize to various areas of the body, leading to progressive deterioration if not promptly identified and treated. The reason for selecting a specific type of cancer for this study was based on the World Health Organization's (WHO) 2020 cancer statistics, provided in Table 1, illustrating the global impact of various cancers.

Cancers like leukemia, myeloma, and lymphoma can impact RBCs, bone marrow, and the lymphatic system. Although these tumors exhibit certain commonalities, their natural histories diverge markedly. Myeloma and lymphoma, which frequently exhibit prolonged disease development, may not display unique heamatological morphological characteristics in blood smears during the initial symptomatic phases [7]. Conversely, characteristic signs of both disorders are more frequently seen in bone marrow or lymphatic tissue, respectively.

The diagnosis process for heamatological malignancies is complex and extends beyond microscopic blood analysis. Initial assessments may include the identification of abnormally produced WBCs via microscopic inspection, but a conclusive diagnosis necessitates a combination of advanced procedures. This encompasses flow cytometry for immunophenotyping, molecular marker analysis, and cytogenetic research, all of which enhance the holistic understanding of the disease. Moreover, while morphological examinations may occasionally utilize image analysis, they often constitute a component of a more comprehensive diagnostic framework rather than an independent procedure [8].

**Figure 3:**
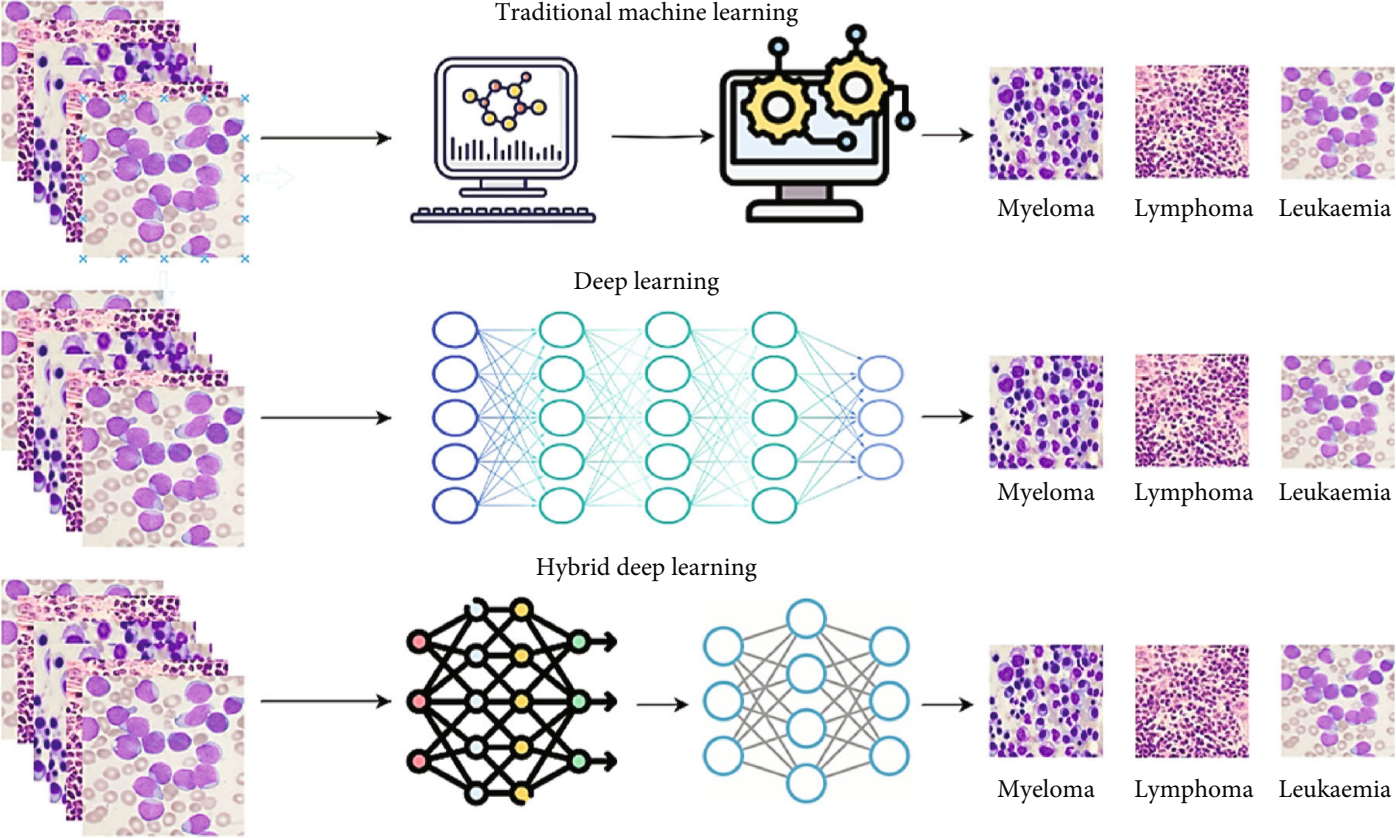
Visual representation illustrating the distinctions between machine learning, deep learning, and hybrid deep learning techniques for blood cancer classification.

Finding innovative cancer detection and treatment methods is a major oncological challenge. One way to diagnose blood cancer is to conduct a complete blood count and examine the WBCs in peripheral blood specimens (NCI 2018). Identifying cancerous cells in blood samples is crucial for making a diagnosis and improves prognosis. Human test outcomes on microscopic blood samples might be subjective due to the concerned individual's experience, maturity, state of mind, exhaustion, etc. Thus, a computerized and intelligent system can help us in avoiding errors in crucial instances. In our study, machine learning (ML), deep learning (DL), and hybrid learning, as represented in Figure 3, are being used to identify blood cancer in microscopic images. Several aspects explain their importance which are listed below:

1. Improved accuracy: ML and DL algorithms can learn from massive datasets to detect cancerous cells more accurately. They can spot hidden data trends that improve diagnosis [9].
2. Automating analysis: ML-automated analysis of images decreases pathologists' manual burden, speeding the processing of samples and diagnosis that improves the treatment of patients [10].
3. Better uncommon subtype detection: DL methods, especially those utilizing a convolutional neural network (CNN), can detect uncommon blood cancer subtypes that are hard to diagnose. Researchers also found that CNNs can categorize lung cancer subtypes on histopathology slides [11].
4. Efficiency: ML models exhibit potential for evaluating unstained digital blood pictures; nonetheless, this skill remains unachieved. Contemporary techniques such as CellaVision continue to depend on stained images, as Romanowsky staining is crucial for observing cell shape and content using light microscopy [12].
5. Data integration: DL models may incorporate genetic, demographic, clinical, and imaging data to enhance blood cancer diagnosis [13].
6. Early detection and monitoring: ML and DL can discover blood cancer early, which is crucial for therapy. They can also track illness development by analyzing successive photos. Bejnordi et al. [14] found that some breast cancer detection algorithms performed as effective as the pathologist's study.
7. Personalized medicine: ML and DL forecast individual reactions to cancer-fighting therapies according to microscopic image patterns, highlighting ML's potential in personalized medicine [15].

The author of [16] and [17] asserted that ML has become a popular kind of computer vision technique, which has encouraged several researchers to focus on studying medical images using this method. After the AlexNet model that took first place in 2012, DL began to gain significant traction. According to Meijs et al. [18], DL employing CNN is now widely used in medical image analysis. The researchers mainly focused on healthcare image analysis in the areas of image classification, object recognition, segmentation, registration, and other relevant topics in particular. There were also neural, ocular, pulmonary, digital pathology, breast, cardiac, abdominal, and musculoskeletal domains where DL was put to use. The diagnosis of various phases of leukemia involves six steps of image processing, which are addressed in the discipline of DL: (1) obtaining images, (2) preprocessing images, (3) segmenting images, (4) extracting features, and (5) identifying cells. The alternative approach leverages standard AI techniques and image processing operations, along with data processing methods such as *K*-means clustering, watershed segmentation, support vector machines (SVMs), fuzzy logic, and *C*-means clustering [19]. In this approach, the convolutional layers in DL capture localized features like colors, endpoints, curves, and oriented edges. As these layers progress deeper, the localized features are amalgamated into larger structural elements such as circles, ellipses, or specific shapes and patterns. Subsequently, these structural or pattern features serve a high-level semantic model representing the feature abstractions of each category. To reduce the dimensionality of the features obtained from convolutional layers, feature downsampling is performed in pooling layers using either mean or max pooling techniques [20]. Conversely, fully connected layers utilize the features extracted from convolutional layers as inputs to function as a classifier, commonly known as a multilayer perceptron (MLP). These densely connected layers capture the spatial relationships within semantic data and convey the co-occurrence aspects of patterns or objects.

In this survey study, we will briefly explain different types of hematologic malignancies with the screening procedures for each. Finally, we examined the potential uses of DL for each cancer kind, including feature extraction, detection, segmentation, prediction, and classification. Given these challenges in hematologic malignancy detection, it is crucial to explore advancements in AI methodologies. The following section outlines the significance of this research and highlights key areas where improvements are needed.

## 2. Research Significance

Hematologic malignancy diagnosis is still hampered by a lack of established evaluation benchmarks and a lack of large, diverse datasets, which together prevent model generalization and repeatability despite notable advancements in AI-driven medical imaging. Furthermore, transformer-based and hybrid architectures are understudied in this field because the majority of research focuses on CNNs. By providing a thorough comparison of conventional ML, DL, and hybrid approaches across leukemia, lymphoma, and myeloma and using a common evaluation framework (accuracy, precision, recall, and area under the curve [AUC]) on benchmark datasets, this review fills these gaps. Our work lays the groundwork for more reliable, clinically applicable diagnostic tools by showcasing cutting-edge architectures like vision transformers and attention-augmented CNNs and stressing the integration of multimodal clinical data, semisupervised learning, and stringent external validation.

Many studies have investigated various AI techniques for hematologic malignancy identification in order to address these issues. The next section examines current approaches, contrasting their advantages and disadvantages.

## 3. A Comparative Analysis of Different Methods Used in the Classification of Leukemia

**Figure 4:**
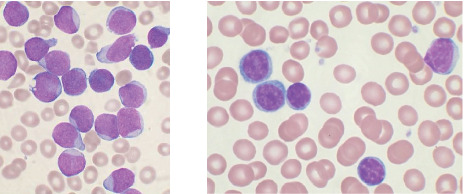
Blood samples as seen under a microscope, showing acute lymphoblastic leukemia (ALL) and nonacute lymphoblastic leukemia (NALL), taken from the ALL_IDB1 and ALL_IDB2 databases [23].

Leukemia is a special class of blood cancer that mostly targets WBCs, which are the blood cells found within bone marrow. There is a sponge-like substance called bone marrow that is present inside the bones [21]. The production of blood cells is disrupted in leukemia, which can result in an abnormally high or low number of blood cells that are not functioning as they should. The disease known as leukemia is caused by the uncontrolled division of young blood-forming cells, which are characterized by excessive blood cell growth that mainly impacts the WBCs (leukocytes) detected in the bone marrow [22]. As shown in Figure 4, the irregular WBCs developed by the bone marrow are the source of leukemia. According to the development rate and the cell line of origin, leukemia may be roughly divided into two categories, one of which is chronic and the other of which is acute, based on the patient's rate of development. These groups were classified according to the rate at which the disease advances and the amount of time it takes for it to achieve its peak progression. In contrast to acute leukemia, chronic leukemia mostly affects adults and is often cured at a moderate pace.

Based on the information that was reported in the year 2015 [24], there were roughly 876,000 people who were affected by acute lymphoblastic leukemia (ALL) globally, and it was responsible for 111,000 fatalities. In the last 50 years, there have been substantial breakthroughs in the treatment of ALL. Timely evaluation and care have demonstrated a substantial enhancement in results, with 5-year survival rates attaining as high as 70% in some instances. In juvenile patients with B-cell acute lymphoblastic leukemia (B-ALL), full remission rates may reach 90% in cohorts from affluent nations [25]. Thus, prompt diagnosis and efficient treatment are essential for enhancing patient outcomes. Medical professionals employ morphology to evaluate the presence of malignant B-lymphoblast cells in the bone marrow for the diagnosis of ALL. Bone marrow aspiration frequently serves as the initial technique for assessment, supplemented by other diagnostic instruments. Quantitative analysis of blood samples can be conducted via computer-aided diagnosis (CAD) systems, which employ ML or DL methodologies to improve accuracy and efficiency [26].

### 3.1. ML Research Innovations

ML techniques are widely used to identify leukemia. Initial processing, feature extraction, and classifications are the typical phases of such approaches. On the other hand, segmentation and feature selection approaches are also used by certain systems to enhance performance even further. For instance, in order to automate the recognition of ALL, Singhal and Singh [27] used a method based on SVMs. The use of local binary patterns (LBPs) to extract geometric and textural information aids the completion of diagnostic tasks. When compared to geometric features, which provide an accuracy of 88.79%, texture features show an even higher level of accuracy at 89.72%, according to the testing results of their suggested approach. In medical terminology, accuracy denotes the capacity of a procedure to accurately categorize situations as positive or negative. This demonstrates the efficacy of the computational method utilizing geometric and texture elements in picture analysis, rather than the diagnostic precision of clinical tests, which entails a more comprehensive assessment. When it comes to video compression and broadcasting, YCbCr is the color space of choice. For better data handling and video reproduction quality, it divides the luminance (luma) and chromatic (color) information. Zare et al. [28] have suggested an approach, wherein each microscopic image's color space is converted to YCbCr, and then, the values of Cb and Cr are obtained according to a Gaussian distribution. In order to train the classifier, further steps involve computing several sets of characteristics, such as morphology, texture, and size. Using random forest as a classifier, their technique achieved 94.3% accuracy in detecting myeloma, two types of leukemia (ALL and acute myeloid leukemia [AML]), and other malignancies [28]. Using microscopic images of the marrow of the bones and blood specimens with pigmentation, Mohapatra et al. [29] proposed a system for screening for acute leukemia. Classification training for an ensemble-based model follows feature extraction from microscopic images. The proposed ensemble outperformed other conventional classifiers like naïve Bayesian (NB), *K*-nearest neighbor (KNN), a radial basis functional network (RBFN), MLP, and SVM with an observed accuracy of 94.73% and normal precision and sensitivity measures above 90%. Afterwards, Patel and Mishra [30] implemented *K*-means clustering for leukemia detection in their unsupervised learning framework. This is useful for estimating the likelihood of leukemia detection by computing the percentage. Using watershed transforms followed by morphological transformations for segmentation, Wang and Orchard [31] proposed a method for ALL detection. They adapted the Gaussian mixture model (GMM) and binary search tree (BST) to classify the data after the extraction of various morphological characteristics. Their proposed method demonstrates an accuracy rate of 95.56% [32]. The discrete orthogonal Stockwell transform (DOST) was used to extract features from images of blood samples for leukemia utilizing linear discriminant analysis (LDA) [33].

### 3.2. DL Research Innovations

Numerous studies have adopted DL techniques to build models for automated leukemia classification. Traditional standalone DL models and learning-based techniques are two subcategories of these DL methodologies [34–36]. As an example, to detect AML [37, 38] using blood sample images, Shaheen et al. [39] proposed an AlexNet-based DL model. Using metrics like accuracy, quadratic loss, recall, and precision, they evaluated their method's performance against that of the LeNet-5 model. In addition to correctly classifying 87.4% of the microscopic images, their suggested approach demonstrates 98.58% accuracy. For the purpose of detecting leukemia, Rehman et al. [40] proposed a CNN design that includes several convolutional and max pooling layers. All microscopic samples undergo preprocessing to convert them to HSV (hue, saturation, value) color space, followed by segmentation to acquire the necessary region of interest, before being fed into the CNN algorithm. Their study found a 97.98% success rate in detecting leukemia. To identify the most important characteristics of leukemic cells, Zakir Ullah et al. [41] proposed an attention-based DL model. Basically, VGG-16 was one of the pretrained DL models on which other models were built on. They validate their technique utilizing a sevenfold cross-validation, and it uses segmented images of normal and leukemic cells. The ConvNet-CNN model proposed by Bodzas et al. [42] can identify ALL and all of its subtypes. The total number of samples in the collection was 363, and they used two different types of datasets. In order to classify leukemia into six distinct groups, Shafique and Tehsin [43] developed a DL model. During training, for effective utilization of time, they used a pretrained AlexNet to perform binary classification on 368 images. Using a combination of transfer learning and DL, [43] also developed a WBC classification system, in which they extracted features from the dataset after doing the necessary preprocessing. Stage three involves running the classification algorithm via the Inception and ResNet models. In their study, they validate the model's reliability using 352 images in total. WBC leukemia was also efficiently classified by Sahlol et al. [44]. They employed VGGNet for deep feature extraction and swarm optimization for simplicity. In order to optimize the deep characteristics for dependable and accurate classification, a bioinspired optimization method is crucial. Bibi et al. [45] conducted one of the most recent studies on leukemia detection. They suggested a structure based on the Internet of medical things (IOMT) [46] with the use of cloud computing and diagnostic equipment linked through Internet-based resources. As a result of the setup of the system, healthcare operators and specialists can detect and treat leukemia patients, which might save workload for both parties. They built their automated system using DenseNet-121 and ResNet-34, two types of CNNs [47, 48]. The suggested method's accuracy was verified on two separate benchmark datasets, LL-IDB and ASH image bank, and the outcomes that have been presented are outstanding. Loey et al. [49] proposed using transfer learning in two separate classification models for leukemia-free and leukemia-affected blood microscopic images. The first model employs decision tree (DT), linear discriminant (LD), SVM, and KNNs as classifiers in addition to a pretrained CNN named AlexNet for the extraction of discriminatory features. The SVM classifier has proven to be more effective through experiments. The second model employs AlexNet for the purpose of feature extraction and classification. As part of their two-stage approach, Liu and Long [50] utilized two inception ResNets, with pretrained ImageNet weights. In the following stage, they used the ALL dataset of human epidermal melanocyte (HEM) cells to fine-tune the models that were trained during the first stage.

### 3.3. Hybrid Model Research Innovations

In order to diagnose leukemia, researchers have developed hybrid DL systems in addition to using standalone models based on DL. To perform automated cell identification, Yu et al. [51] suggested a hybrid approach that makes use of ResNet50, VGG-16, and VGG-19, all of which rely on cutting-edge CNNs. A number of standard ML methods, including DT, SVM, KNNs, and logistic regression (LR), are contrasted with the suggested strategies that achieved 88.50% cell recognition precision. Shafiq and Gu [52] also developed a DL-based hybrid model with dual CNN architectures to improve performance accuracy [53] which shows 89.70% precision when tested on 636 blood specimens containing healthy and ALL cells. Additionally, Mourya et al. [54] used a ViT–CNN, also called a vision transformer CNN based on ensemble learning. Jiang et al. [55] suggested a method that can differentiate between normal and cancer cells, which aids in the diagnosis of ALL. Their technique incorporates a vision transformer and a model built on CNN to enhance classification results by drawing characteristics from a large number of cells in different ways. To get over the problems caused by the imbalanced dataset, they have additionally improved the data enhancement–random sampling (DERS). With a remarkable success rate of 99.03%, their suggested algorithm demonstrates that it is a viable CAD system for ALL. Kassani et al. [56], in their hybrid technique, utilized VGG-16 and MobileNet to classify leukemic lymphoblasts after extracting deep characteristics from them. They achieved a sensitivity level of 95.17%, a precision level of 98.58%, and an accuracy of 96.17%. In addition, Zoph et al. [57] used the NASNetLarge and VGG-19 DL models to classify healthy B-lymphoid cells as well as leukemic B-lymphoblast tissues, and the resulting detection performance was 96.58%. They demonstrated that ensemble learning is superior to an individual model and that their suggested model successfully identifies ALL [58]. In order to identify acute lymphocytic leukemia from images of blood samples, Jha and Dutta [59] presented a DL model built on the chronological sine cosine algorithm (SCA). As an example, Tan and Shi [60] achieved a 99.66% accuracy value in diagnosing acute lymphocytic leukemia by using generative adversarial optimization (GAO) [61]. In a similar vein, Mirjalili [62] used artificial neural networks (ANNs) and genetic algorithms (GAs) to segment ALL based on local pixel information; they reported a precision of 97.07% [63]. A 98.60% successful diagnosis for ALL was produced by Acharya and Kumar [64] using data mining and image segmentation. Bukhari et al. [65] use a DL architecture that takes use of squeezing and excitation learning to identify leukemia in blood samples that are extremely small. Their method improves the capacity to distinguish between regular and leukemic cells based on their features by highlighting the interdependencies across channels at all characterization levels. When it comes to identifying leukemia from microscopic images, the algorithm outperforms typical DL techniques. Tests on two open-source datasets, ALL_IDB1 and ALL_IDB2, yielded a 98.3% success rate. Hariprasath et al. [66] performed cell identification and classification using the YOLO V4 (You Only Look Once) algorithm. The approach was tested on two standard datasets, ALL_IDB and CNMC, and the results showed an average precision of 96.06% and 97.8%, respectively. When compared to more conventional ML techniques, the optimized DCNN suggested by Kumar et al. [67] for leukemia classification outperformed the baseline methods, with an accuracy rate of 97.8%. According to Bibi et al. [68], who first suggested DenseNet and ResNet for leukemia detection, ResNet-34 and DenseNet-121 outperform all prior methods in terms of diagnostic power. Methods for two-stage pipelining utilizing transfer learning as well as semantic segmentation were proposed by Raina and Gondhi [69]. Using the entire blood smear microscopy images, they classified five types of leukocytes in peripheral blood using DeepLabv3+ and AlexNet to perform leukocyte segmentation.  Table 2 shows a summarized comparison of different approaches with the methods discussed earlier.

## 4. A Comparative Analysis of Different Methods Used in the Classification of Lymphoma

Lymphoma refers to a type of immune system–related hematologic malignancy. It targets lymphocytes, a kind of WBC that plays a crucial role in the immune system [70], which starts in the lymphatic system, when the formation of lymphocytes goes out of control. This uncontrollable proliferation of lymphocytes is termed lymphoma [71].

**Figure 5:**
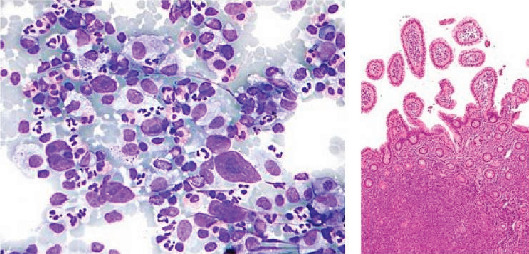
Samples of Hodgkin's lymphoma and non–Hodgkin's lymphoma [72].

The two most common varieties of lymphoma are non–Hodgkin's lymphoma (NHL) and Hodgkin's lymphoma samples as shown in Figure 5. The most frequent kind is diagnosed in about 14,000 individuals annually in the United Kingdom [73]. This study compares the efficacy of radiomic characteristics used to distinguish follicular lymphoma (FL) from diffuse large B-cell lymphoma (DLBCL) in FDG PET/CT images processed by ML techniques.

### 4.1. ML Research Innovations

With 136 radiomic attributes, gradient boosting outperformed the other two tree-based ensemble classification methods (XG Boosting plus AdaBoosting) using LR. Gradient boosting attained a precision of 80% and an AUC of 0.86 [74, 75]. This method outperformed a LR framework that relied on SUVmax, suggesting that radiomic characteristics had diagnostic relevance beyond only using SUVmax to differentiate between FL and DLBCL.

### 4.2. DL Research Innovations

Yu et al. [76] developed a model that classified primary intestinal T-cell lymphomas into two types: monomorphic epitheliotropic intestinal T-cell lymphoma (MEITL) and intestinal T-cell lymphoma not otherwise specified (ITCL-NOS). The model uses morphological characteristics to achieve this. A deep neural network (DNN) was trained on a dataset consisting of 40 samples of histopathology, the model was able to identify and separate lymphocyte nuclei, and it calculated quantitative nuclear morphometrics. Utilizing these characteristics as its basis, the XGBoost algorithm attained a remarkable accuracy of 96%, demonstrating its high level of accuracy in classification. By analyzing cellular shape, this human-interpretable technique shows how ML may improve lymphoma diagnosis precision. In contrast, Li et al. [77] achieved a 100% success rate in classifying pathologic images using several CNNs to distinguish between non-DLBCL and DLBCL in humans. The tedious procedure, expertise, and aggressive cell heterogeneity make clinico-morphological feature–based lymphoma diagnosis problematic. It was needed to improve the treatment for lymphoma patients and provide them with high-quality care by using AI-based models, including CNNs using pattern detection algorithms [78]. With the use of efficient Net CNNs, histologic images of NHL patients were classified with a high degree of accuracy (95.6%) [79]. A DL approach for identifying and categorizing lymphoma was disclosed by Miyoshi et al. [80], where they gathered hematoxylin and eosin (H&E)-marked whole-slide images (WSIs) of individuals suffering from BCL, FCL, and lymphoid hyperplasia. When compared to the average pathologist-recorded accuracy (76%) using WSIs, the method's 97% precision in classifying the three lymphoma kinds is much substantial. The classification of T-cell lymphomas is aided by an ML-based diagnosis. For the purpose of developing a DNN dataset, Xiao et al. [81] gathered 40 histological WSIs. They were able to classify T-cell lymphomas, such as intestinal T-cell lymphoma which is challenging to diagnose morphologically by extracting image features. Similarly, cytochemically stained smears can be used to diagnose lymphoma using a DL-based CNN method. They gathered 128 cases' worth of H&E-stained slides. They also developed a diagnosis model for each of the four kinds of lymphomas—small lymphocytic lymphoma, benign lymph node, DLBCL, and Burkitt lymphoma using WSIs. As a result, they were able to use the CNN model to accurately discern between the various lymphoma kinds from H&E-stained images 95% of the time [82].

### 4.3. Hybrid Model Research Innovations

To differentiate FCL with follicular hyperplasia, Syrykh et al. [83] used a CNN-based method known as Bayesian neural networks (BNNs). Their total accuracy in detecting FCL using H&E-stained lymph node slides was 91%. Additionally, unusual lymphoma forms including mucosa-associated lymphoma tissue (MALT) were detected and classified using AI models. Federated gradient boosting trees (FGBTs), federated gradient boosting trees with dropouts (FDARTs), federated multilayer perceptron (FMLP), and federated multinomial naïve Bayes (FMNB) are some of the federated AI techniques that Pezoulas et al. [84] created. The FDART had an 82.8% success rate in detecting and classifying MALT lymphoma in individuals with primary Sjogren's disease. Shankar et al.'s [85] approach utilizing ML applying LymphoML to H&E-stained slides allows the prediction of lymphoma subtypes. The *F*1-score, which is a measure of accuracy and sensitiveness, helps to predict many forms of lymphomas, such as DLBCL (78.7%), classic Hodgkin's lymphoma (74.5%), and mantle cell lymphoma (MCL) (71.0%). With a diagnosis accuracy of about 85%, the technique outperformed the competition when taking nuclear shape factors into account. Therefore, the procedure was shown to be both convenient and error-free following external validation, suggesting that it might be used in clinical practices. In future developments of AI-based diagnosis employing immunohistochemistry (IHC) in hematologic neoplasms, it is important to incorporate patients' clinicopathology, as this algorithm may not account for it [86]. When comparing gene expression patterns to IHC, ML correctly differentiates DLBCL. There is a 91.6% success rate for using ML to distinguish between germinal center and nongerminal center subtypes of DLBCL, according to Da Cost [87]. In a similar vein, Carreras et al. [88] used AI-based ANNs to detect MCL prognostic indicators. Using ANNs, they examined 123 cases and found 58 genes that accurately predicted survival for MCL patients (AUC: 0.9). They found that RGS1, which stands for regulator of G-protein signaling, was linked to a poor prognosis when looking at IHC-based prognostic indicators. A clear comparison of different approaches is shown in Table 3.

## 5. A Comparative Analysis of Different Methods Used in the Classification of Myeloma

**Figure 6:**
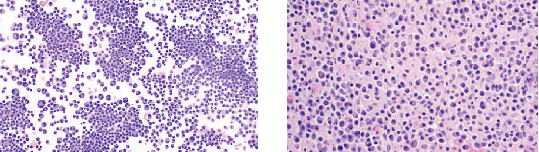
Samples of multiple myeloma cancer cells.

Plasma cells are the WBCs that are affected by myeloma. Under normal circumstances, plasma cells produce antibodies that aid in the defense against infections [89]. When plasma cells develop myeloma, they begin to produce antibodies that are not functioning as they are supposed to be. It is also the case that these aberrant cells proliferate at a faster rate. The fact that myeloma affects more than one part of the body is the reason why it is frequently referred to as multiple myeloma. The samples are depicted in Figure 6. Despite the fact that there are several subtypes of myeloma, the treatment for each subtype is the same [90]. The fact that they do not exhibit any signs of myeloma at the time of diagnosis indicates that they may not require therapy for a considerable amount of time [91].

While some people are diagnosed with myeloma at a much earlier age, the majority are men, and most of them are over the age of 70. It is possible that the lag in the diagnosis will cause the therapy to be delayed, which will then lead to the start of serious issues [92]. An increased tumor strain and the beginning of organ damage both contribute to a lower survival rate [93]. As a result of this, there has been a growing interest in the use of AI approaches for the detection of MM.

### 5.1. DL Research Innovations

When taking into account the capability of the gradient boosting decision tree (GBDT) to handle issues pertaining to diagnosis and categorization, it is regarded as a genuine ensemble learning system by Hastie et al. [94]. It was determined that a total of 4187 blood and biochemical examinations were gathered. These examinations included 1741 results from patients with myeloma as well as 2446 data gathered from persons who did not have myeloma. The GBDT, SVM, DNN, and RF algorithms were utilized in the development of an assisted diagnostic model for MM. The receiver operating characteristic (ROC) curve was utilized in order to make comparisons between the various approaches. All of the ML algorithms that were tested, including RF, DNN, and GBDT, performed without any problems. With regard to the patients with MM, the GBDT demonstrated the greatest levels of accuracy (92.9%), replicability (90.0%), and *F*1-score (0.915). After performing the calculation to determine the maximized area within the ROC, it was concluded that the findings of GBDT (AUC: 0.975; 95% confidence interval (CI): 0.963–0.986) were superior to those of SVM, DNN, and RF. As a result, the method that is based on ML, which is fed by conventional laboratory data, is able to accurately diagnose MM, which increases the probability of early detection [95].

### 5.2. Hybrid Model Research Innovations

Vyshnav et al. [96] investigated the performance of DL-based segmentation algorithms, especially Mask-RCNN and U-Net, in identifying multiple myeloma cells from bone marrow aspirate slides. A total of 85 microscopic images consisting of five individuals who have been diagnosed with multiple myeloma are included in the dataset that was retrieved from the Cancer Imaging Archive (CIA). Manual annotation was performed on these samples with the help of the VGG Image Annotation programme. According to the findings of the research, the Mask-RCNN method performed better than the U-Net model. It achieved greater levels of accuracy, precision, recall, and *F*1-score metrics. In particular, the Mask-RCNN model demonstrated an accuracy of 93.99%, while the U-Net model achieved an efficiency of 89.62%. Wang et al. [97] utilized U-Net and Faster RCNN models in order to properly analyze CT images for the purpose of myeloma diagnosis. The segmentation of CT scans and the labelling of lesions were the primary focuses of this study, which included 186 individuals who were suspected of having myeloma. The results of the study demonstrated the effectiveness of DL models in lesion segmentation and classification, with a classification accuracy rate that reached as high as 99%. Allegra et al. [98] used an ensemble DL–based technique that is presented for the autonomous binary classification of cancerous myeloma histological samples. The approach makes use of pretrained models VGG-16, VGG-19, and ResNet-50, and it also incorporates transfer learning. Through the achievement of high prediction accuracies of 99.77%, 97.92%, and 97.40%, respectively, the research illustrates the efficacy of these algorithms in enhancing the early diagnosis of individuals suffering from multiple myeloma. Through the use of sophisticated image classification methods, this methodology highlights the essential role that DL plays in improving diagnostic procedures for complicated illnesses such as multiple myeloma. The different approaches are clearly compared in Table 4.

## 6. Evaluation Tools

AUC and ROC curves are two of the tools used to justify the models' performance in this study. These visual aids allow for a comparison of the model's efficacy visually by plotting the true-positive rate versus the false-positive rate. A better-performing model is indicated by a higher AUC value, especially when it comes to differentiating between cancerous and noncancerous blood cells.

Furthermore, the number of true positives, true negatives, false positives, and false negatives is shown using confusion matrices, providing a clear breakdown of the model's performance in every scenario. These techniques shed light on possible areas for model enhancement, like lowering false positives in important applications for cancer detection.

Learning curves and precision–recall curves are two examples of visualisation techniques that are useful in demonstrating the effectiveness of various models at different phases of testing and training. Models' ability to manage class imbalances is assessed with the aid of precision–recall curves, which illustrate the trade-off between precision and recall. Learning curves show how the model learns over time and make it possible to identify instances of overfitting or underfitting when training.

These technologies offer empirical support for statements made about the superiority of DL models, including CNNs and hybrid models, which frequently outperform traditional ML techniques in picture classification tasks linked to the identification of hematologic malignancies. These outcomes show how successful our strategy is. Their importance, comparisons with current approaches, and possible directions for future research are covered in detail in the next section.

## 7. Discussions and Challenges

**Figure 7:**
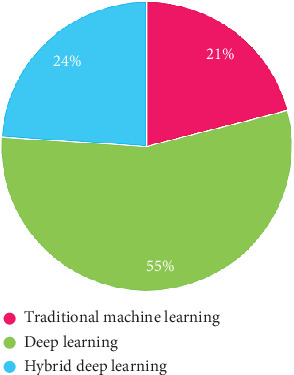
Overview of diverse deep learning methods employed in research studies.

Every strategy investigated and referred here has its own unique benefits and challenges. Traditional ML methods may not be able to handle complicated data without considerable feature engineering while being more interpretable and requiring less resources. When used in place of automatic feature learning, DL techniques—in particular, CNNs—provide a potent substitute that significantly enhanced classification tasks. Nevertheless, they demand sizable computing resources and annotated datasets. By combining the greatest features of both worlds, hybrid models seek to improve generalizability and model performance as in Figure 7. Lymphoma diagnosis, distinction, risk stratification, and prognostic marker discovery are areas where AI-based algorithms fall short. At this time, no ML algorithm for lymphoma diagnosis has been approved by the FDA. Further evaluation of studies on large-scale datasets, especially those pertaining to uncommon conditions, is necessary for the development of a cheap and more accurate ML-based diagnosis algorithm that can enhance healthcare. Some lymphomas, such as enteropathic T-cell lymphoma and ocular MALT lymphoma [84], might serve as examples. Due to the tiny sample size and AI's incapacity to obtain the global solution, the system fails under these conditions and prefers to be trapped in local minima.

In terms of evaluation metrics, the performance of the models is evaluated based on a set of predefined criteria, with a particular emphasis on the following.

### 7.1. Accuracy

Utilizing this statistic, one may determine the percentage of instances that have been accurately classified across all categories and cases. It is essentially a reflection of the model's capacity to accurately diagnose individuals who have blood cancer and to correctly identify the benign samples. When the accuracy value is greater [99–102], it suggests that the model is more exact. The formula for accuracy is presented in Equation (1):

(1) $$\begin{array}{c} \text{Accuracy}=\frac{\text{TP}+\text{TN}}{\text{TP}+\text{TN}+\text{FP}+\text{FN}}, \end{array}$$

where TN stands for true negative, indicating cases correctly identified as negative; TP denotes true positive, referring to cases accurately recognized as positive; FP signifies false positive, which are instances incorrectly labelled as positive; and FN represents false negative, highlighting cases that are wrongly classified as negative.

### 7.2. Precision

This statistic determines the proportion of cases that are actually positive out of the total number of positive forecasts [103]. To be more specific, precision refers to the accuracy of the model in properly identifying individuals who truly have any type of blood cancer. This is specifically relevant to the diagnosis of blood cancer. The mathematical formula for accuracy is defined as stated in Equation (2):

(2) $$\begin{array}{c} \text{Precision}=\frac{\text{TP}}{\text{TP}+\text{FP}}. \end{array}$$

### 7.3. Recall

It is a measure used to evaluate the efficacy of the model in reliably identifying individuals with leukemia illness out of all of the real instances of blood cancer. The capability of the model to capture all relevant occurrences of the illness is evaluated, with the goal of ensuring that individuals with different types of blood cancer are appropriately identified. Recall is determined by dividing the total number of true-positive identifications by the sum of true positives and false negatives. This computation is used to determine the percentage of genuine blood cancer cases that the model is able to correctly identify. The formula for recall is represented in Equation (3):

(3) $$\begin{array}{c} \text{Recall}=\frac{\text{TP}}{\text{TP}+\text{FN}}. \end{array}$$

### 7.4. *F*-Score

The *F*-score is a comprehensive measure that analyses the overall performance of the model by carefully integrating the values of accuracy and recall in a way that is harmonious. The purpose of this metric is to provide a balanced assessment that takes into account both the precision of positive predictions (precision) and the capacity of the model to recognize all instances that matter (recall). This metric is especially helpful for evaluating the trade-offs between accuracy and recall since it provides a single score that shows the model's capacity to effectively identify instances while minimizing the number of blood cancer cases that are overlooked or incorrectly diagnosed. In order to provide a comprehensive analysis of the diagnostic capabilities of the model, the *F*-score is computed using a method that incorporates both the precision and recall values as in Equation (4):

(4) $$\begin{array}{c} F1=2.\frac{\text{precision}.\text{recall }}{\text{precision}+\text{recall}} \end{array}$$

There are a number of problems with datasets that might have an impact on the performance and generalizability of these models in the current context of blood cancer classification utilizing DL and ML models. The following is a list of the most important problems observed:

a. Restricted size and availability: There are not many large, diversified datasets available in the medical industry, especially when it comes to blood cancers. The delicate nature of medical data, patient privacy issues, and the difficulty of getting thorough clinical annotations are the main causes of this paucity.
b. Data privacy and confidentiality: Strict rules on the collection, usage, and sharing of patient data are enforced by privacy legislation; these limitations limit access to medical images required for the training and validation of blood cancer classification models [104].
c. Unbalance in dataset: Class imbalance is a common problem in many blood cancer datasets, where some blood cancer types are underrepresented in comparison to others. Prediction accuracy for less frequent cancer types is impacted by models that are biassed towards the dominant class as a result of this imbalance [105].
d. Image quality and variability: The consistency of datasets impacted by the variations in imaging techniques (such as staining methods and types of microscopes) as well as the quality of medical images. DL model training becomes more challenging due to noise introduced into the data by varying image quality and acquisition conditions [106].
e. Absence of annotations: The process of annotating medical photographs is expensive and time-consuming, requiring specialized skills. As a result, the possibility of sophisticated model training is limited because many datasets do not have comprehensive annotations. Figure 7 shows the overview of different DL methods employed in our study.

f. Enhancing generalization in trained predictive models: Creating a model that is good at learning and generalizing is the hardest part of creating a system for the identification and categorization of blood cancer. Strong generalization skills in a model necessitate an advanced learning strategy that can make use of both randomly assigned and real labels. It also necessitates training techniques that improve computing performance and strong approaches to effectively use the resources that are accessible. DNN's advanced feature extraction capabilities have led to significant success in medical image analysis [107], particularly in the categorization of cancer. Surprisingly, these networks frequently perform well in terms of generalization, even in cases when there are many more model parameters than training data. Merely minimizing training mistakes is insufficient in some cases though as an incorrectly selected global minimum might have a negative impact on the model's generalization capacity. Generalization is slightly impacted by the training error minimization strategy [108].
g. Real-world circumstances: Presently available DL techniques are not reliable enough to be implemented in real-world healthcare diagnostic systems without expert medical advice. It needs domain specialists' insights in addition to technical expertise to develop learning models for blood cancer classification and medical image analysis. Investigating technologies that are accurate and trustworthy enough to function without the assistance of medical professionals and that can be incorporated into actual healthcare settings is crucial.
h. Computational time: DL is used in most algorithms that are used for the diagnosis of blood cancer since it performs better than other techniques. However, employing DL architectures like CNNs or R-CNNs for the interpretation of high-resolution medical images is memory- and time-intensive. The number of layers that these methods include in their CNNs directly relates to their level of complexity [109].

These results clearly show that including AI-driven models can improve the identification of hematologic malignancy. Along with potential avenues for further research, key contributions are outlined in the final section.

## 8. Conclusion and Future Directions

The identification and classification of haematological malignancies remain a significant challenge in medical diagnostics, requiring precise and efficient methodologies to support early detection and treatment decisions. This study systematically reviewed and analysed various ML and DL techniques applied to the classification of haematological malignancies, focusing on leukemia, lymphoma, and multiple myeloma. The findings indicate that DL models, particularly CNNs and hybrid architectures, consistently outperform traditional ML approaches in terms of accuracy, sensitivity, and robustness. While classification accuracies exceeding 95% have been reported on benchmark datasets, challenges such as limited dataset availability, class imbalance, and a lack of clinical validation persist, necessitating further advancements in this area.

A key contribution of this work is a detailed comparative analysis of different AI methodologies, highlighting their advantages and limitations. Traditional ML models, such as SVMs and DTs, have demonstrated effectiveness in structured data analysis but struggle with high-dimensional and complex imaging data. In contrast, DL architectures, particularly those leveraging transfer learning and attention-based mechanisms, have shown superior feature extraction capabilities, improving classification performance. Hybrid models combining CNNs with ensemble learning or attention-based techniques have demonstrated potential in further enhancing diagnostic accuracy while minimizing overfitting.

Despite these advancements, several challenges must be addressed to facilitate clinical adoption. First, the scarcity of large, annotated datasets limits the generalizability of existing models. Techniques such as data augmentation, generative adversarial networks (GANs), and federated learning can help mitigate this issue. Second, the inherent class imbalance in haematological malignancy datasets affects model performance, necessitating advanced sampling strategies and cost-sensitive learning approaches. Third, external validation on diverse, multicentre datasets is crucial to ensure that AI models perform reliably across different patient populations and clinical settings. Additionally, integrating multimodal clinical data—such as genetic markers, histopathological findings, and laboratory test results—could enhance diagnostic precision and reliability.

Future research should focus on developing explainable AI (XAI) methods to improve interpretability, ensuring that clinicians can confidently utilize AI-based diagnostic tools. Furthermore, regulatory considerations, ethical implications, and patient data privacy must be prioritized to enable real-world deployment.

In summary, while AI-driven techniques have made significant strides in detecting and classifying haematological malignancies, continued research is essential to bridge the gap between experimental success and clinical implementation. By addressing current limitations and leveraging emerging AI technologies, automated diagnostic systems can significantly enhance disease detection, leading to improved patient outcomes and more efficient healthcare solutions.

## Figures and Tables

**Table 1:** Report on global cancer statistics from the World Health Organization's Global Cancer Observatory for the year 2020 [6].

**Cancer type**	**Cases**	**Deaths**
Breast cancer	2.26 million	0.685 million
Lung cancer	2.21 million	1.800 million
Colon and rectum cancer	1.93 million	0.916 million
Prostate cancer	1.41 million	0.250 million
Skin cancer	1.20 million	0.830 million
Stomach cancer	1.09 million	0.769 million

**Table 2:** Comparison with existing works on leukemia.

**Author and references**	**Methods**	**Dataset**	**Accuracy**
Singhal and Singh [27]	SVMs	ALL-IDB2	89.72%
Zare et al. [28]	Color space conversion to YCbCr, Gaussian distribution	ALL-IDB1 and ALL-IDB2	94.3%
Mohapatra et al. [29]	Ensemble-based model	Samples from Ispat General Hospital, Rourkela, India	94.73%,
Mishra et al. [32]	GMM and BST for classification	ALL-IDB1	95.56%
Shaheen et al. [39]	AlexNet-based model vs. LeNet-5	Microscopic images of blood samples for AML	98.58%
Rehman et al. [40]	CNN with preprocessing to HSV	Amreek Clinical Laboratory Saidu Sharif Swat KP Pakistan	97.98%
Bodzas et al. [42]	ConVNet CNN vs. SVM, MLPs, RFs	ALL-IDB1 and ALL-IDB2	89.2%
Shafique and Tehsin [43]	Pretrained AlexNet	ALL-IDB2	92.6%
Sahlol et al. [44]	VGGNet for deep feature extraction	ALL-IDB1	95.3%
Bibi et al. [45]	DenseNet-121 and ResNet-34	LL-IDB and ASH image bank for leukemia detection	97%
Yu et al. [51]	Hybrid approach with ResNet50, VGG-16, and VGG-19	ALL-IDB2	88.50%
Mourya et al. [54]	Dual CNN architectures	636 blood specimens (healthy and ALL cells)	89.70%
Jiang et al. [55]	ViT–CNN based on ensemble learning	ISBI2019	99.03%
Kassani et al. [56]	Hybrid of VGG-16 and MobileNet	ALL-IDB1	96.17%
Zoph et al. [57]	NASNet large and VGG-19	ALL-IDB1 and ALL-IDB2	96.58%
Jha and Dutta [59]	DL model	Acute lymphocytic leukemia from blood sample images	97.3%
Tan and Shi [60]	Generative adversarial optimisation (GAO)	ALL-IDB2	99.66%
Mirjalili [62]	ANN and GA	ALL-IDB2	97.07%
Acharya and Kumar [64]	DL model	ALL-IDB1	98.60%
Bukhari et al. [65]	DL and excitation learning	ALL-IDB1	98.3%
Hariprasath et al. [66]	YOLO V4	ALL DBI and CNMC datasets	ALL DBI: 96.06%, CNMC: 97.8%
Kumar et al. [67]	Optimized DCNN	ALL-IDB1, ALL-IDB2	97.8%
Bibi et al. [68]	DenseNet and ResNet	Not specified	98.06%
Raina and Gondhi [69]	DeepLabv3+ and AlexNet	ALL-IDB2	92.9%

**Table 3:** Comparison with existing works on lymphoma.

**Author and references**	**Methods**	**Dataset**	**Performance metrics**
Zhao et al. [75]	DNN with XGBoost	40 whole-slide histopathology images	96.6%
Yu et al. [76]	Several CNNs	Non-DLBCL vs. DLBCL	100%
Li et al. [77]	EfficientNet CNNs	Non-Hodgkin's lymphoma patients	95.6%
Steinbuss et al. [79]	DL approach	BCL, FCL, lymphoid hyperplasia	97%
Miyoshi et al. [80]	DNN	40 histological WSIs	94.3%
Xiao et al. [81]	CNN	H&E-stained slides of four lymphoma types	95%
El Achi et al. [82]	CNN-based BNN	H&E-stained lymph node slides	91%
Syrykh et al. [83]	Federated AI techniques	Patients with primary Sjogren's disease	82.8%
Pezoulas et al. [84]	ML with LymphoML	H&E-stained slides	*F*1-scores: DLBCL (78.7%), Hodgkin lymphoma (74.5%), MCL (71.0%)
Abdul-Ghafar et al. [86]	ML	Gene expression patterns vs. IHC	91.6%
Da Costa [87]	ANNs	123 cases	90%

**Table 4:** Comparison with existing works on myeloma.

**Author and references**	**Methods**	**Dataset**	**Performance metrics**
Yan et al. [95]	DL-based segmentation with Mask-RCNN and U-Net	85 microscopic images from five individuals with multiple myeloma, TCIA	Mask-RCNN: Accuracy: 93.99%, U-Net: Efficiency: 89.62%
Vyshnav et al. [96]	U-Net and Faster RCNN for CT image analysis	186 individuals	Classification accuracy: 99%
Wang et al. [97]	Ensemble DL with VGG-16, VGG-19, ResNet-50, and transfer learning	Cancerous myeloma histological images	VGG-16: 99.77%, VGG-19: 97.92%, ResNet-50: 97.40%

## Data Availability

Data sharing is not applicable to this article as no new data were created or analyzed in this study.
